# Suppressor of cytokine signaling-3 expression and its regulation in relation to inflammation in Chronic Obstructive Pulmonary Disease

**DOI:** 10.3389/fimmu.2024.1320077

**Published:** 2024-03-12

**Authors:** Mariaenrica Tinè, Elisabetta Balestro, Sara Carpi, Tommaso Neri, Davide Biondini, Maria Conti, Alvise Casara, Nicol Bernardinello, Elisabetta Cocconcelli, Graziella Turato, Simonetta Baraldo, Alessandro Celi, Paolo Spagnolo, Manuel G. Cosio, Marina Saetta, Erica Bazzan

**Affiliations:** ^1^ Department of Cardiac, Thoracic, Vascular Sciences and Public Health, University of Padova, Padova, Italy; ^2^ Department of Health Sciences, University ‘Magna Græcia’ of Catanzaro, Catanzaro, Italy; ^3^ National Enterprise for NanoScience and NanoTechnology (NEST), Istituto Nanoscienze-Centro Nazionale Ricerche (CNR) and Scuola Normale Superiore, Pisa, Italy; ^4^ Centro Dipartimentale di Biologia Cellulare Cardiorespiratoria, Dipartimento di Patologia Chirurgica, Medica, Molecolare e dell’Area Critica, Università degli Studi di Pisa, Pisa, Italy; ^5^ Department of Medicine, University of Padova, Padova, Italy; ^6^ Meakins-Christie Laboratories, Respiratory Division, McGill University, Montreal, QC, Canada

**Keywords:** socs3, extracellular vesicles, COPD, miRNA, human BAL, alveolar macrophages

## Abstract

**Background:**

The family of Suppressor of Cytokine Signaling (SOCS) acts as a controller of the duration and intensity of cytokine function by negatively regulating the JAK-STAT signaling pathway. SOCS’ role in inflammatory diseases in animal models is well demonstrated. However, its role in the development of human disease is still under investigation. SOCS3 plays an important role in tumor development where its downregulation has been implicated in the pathogenesis of various solid tumors such as triple-negative breast cancer.

**Aim:**

The aim of this work was to study (1) the expression of SOCS3 in smokers’ lungs and its relation to the degree of inflammation and (2) SOCS3 regulation by microRNA (miRNA) in alveolar-macrophage (AM)-derived extracellular vesicles (EVs) in bronchoalveolar lavage (BAL).

**Methods:**

Group A: 35 smokers’ [19 with COPD (SC) and 16 without COPD (S)] and 9 nonsmokers (NS); SOCS3, TNFα in AM, and CD8^+^ T cells were quantified by immunohistochemistry, in lung tissue. Group B: additional 9 SC, 11 S, and 5 NS; AM-EVs expressing SOCS3 (CD14^+^SOCS3^+^) and SOCS3 suppressors miRNA-19a-3p and 221-3p in EVs were quantified by flow cytometry and PCR, in BAL.

**Results:**

The percentage of SOCS3^+^ AM was higher in SC [68 (6.6–99)%] and S [48 (8–100)%] than in NS [9.6 (1.9–61)%; *p* = 0.002; *p* = 0.03] and correlated with % of TNFα^+^AM (*r* = 0.48; *p* = 0.0009) and CD8^+^ T cells (*r* = 0.44; *p* = 0.0029). In BAL, the CD14^+^SOCS3^+^ EVs/μL were increased in SC [33 (21–74)] compared to S [16 (8–37); *p* = 0.03] and NS [9 (7–21); *p* = 0.003]. Conversely, miRNA-19a-3p and miRNA-221-3p expression were increased in S when compared to SC [19 (2–53) *vs*. 3 (0.6–8); *p* = 0.03 and 3 (0.005–9.6) *vs*. 0.2 (0.08–0.7); *p* = 0.05].

**Conclusions:**

The suppressor function of SOCS3 in COPD seems to be overridden by other factors and does not follow the animal-model paradigm. Expression of SOCS3 in BAL macrophage-derived EVs might be useful to assess the degree of inflammation and possible progression of COPD. Downregulation of SOCS3, by miRNA, in smokers without COPD might contribute to the risk of developing cancer in these patients.

## Introduction

Cigarette smoking induces Chronic Obstructive Pulmonary Disease (COPD) in approximately 20% of smokers, and the disease, in those who develop it, is mild to moderate in a large proportion of them. The severity of the disease correlates with the degree of adaptive immune inflammation in the airways and parenchyma with features of autoimmunity present in the severe disease (1–3).

This variability in incidence and severity of disease, not related to the amount smoked, indicates that unknown factors controlling the inflammatory response are at play. Cytokines like IL-1, IL-6, IL-18, and IL-12 are key protagonists in the start and maintenance of an inflammatory response, but stringent mechanisms of signal attenuation are essential for ensuring the avoidance of untoward effects like autoimmunity and cancer.

The JAK-STAT pathway is a crucial signaling pathway involved in the transmission of signals from the cell membrane to the nucleus in response to various cytokines and growth factors. The family of Suppressor of Cytokine Signaling (SOCS) proteins plays a crucial role in negatively regulating the JAK-STAT signaling pathway. SOCS proteins act as feedback inhibitors to control the duration and intensity of cytokine signaling (4, 5).

Of the eight SOCS proteins described in mammals, SOCS1 and SOCS3 are the ones predominantly associated with the regulation of inflammatory cytokine receptor signaling by their function as inhibitors for JAKs and their interaction with STAT protein substrates, thereby terminating signal propagation (6). Both SOCS1 and SOCS3 proteins are induced following TLR4 stimulation by LPS and regulate aspects of the TLR signaling. Despite their similarities, there are notable differences between SOCS1 and SOCS3 (7). SOCS1 is induced by a wide range of cytokines, including IFN-γ via STAT1 and those that utilize the IL-2 receptor gamma chain (IL-2Rgc) via STAT5, with its major mechanism of action being to inhibit JAK1 and JAK2 kinases (4). Through these mechanisms, SOCS1 regulates a raft of cytokines central to control of immunity and inflammation, including modulation of IFN-γ. SOCS3, the best-studied SOCS in inflammatory conditions, regulates STAT activation in response to cytokines using the gp130 receptor complex for cytokines belonging to the IL-6 family along with other cytokines, like IL-10, by blocking JAK2 and inhibiting STAT3 activation. Of note, the loss of SOCS3 does not abolish the inhibitory effects of IL-10 on cytokine production from LPS-activated macrophages, which indicates that SOCS3 may specifically control IL-6 signaling (8).

Of interest, alveolar macrophages in cell culture can secrete extracellular vesicles (EVs) for uptake by other cells in an attempt to blunt inflammatory signaling (9). However, only few studies examined the presence of macrophage-derived SOCS3 in EVs from human fluids (10–13), like BAL, and especially their possible role in COPD.

The role played by SOCS3 in human diseases has been investigated mainly in patients with inflammatory bowel disease (IBD) (14–16), where SOCS3 mRNA and protein expression in bowel biopsies correlated with the severity of histological inflammation. These findings contrast with those of the mouse model of IBD, where increased SOCS3 expression seems to limit the extent of inflammation. The differences in the possible role of SOCS3 and of its regulatory miRNAs in animal models and human diseases are striking and not easy to understand. Among the multiple miRNAs implicated in the regulation of SOCS3, miRNA-19a-3p and miRNA-221-3p have been shown to be involved in COPD (17–25).

The study of smoking-induced COPD, where the response to smoking varies from severe COPD to no disease, could be a good human model where the expression of SOCS3 and its association with the degree of inflammation and the severity of disease could be investigated. Furthermore, the study of EVs in BAL and their content of SOCS3, along with the SOCS3 inhibitory miRNAs, 19a-3p and 221-3p, might add to our understanding of the role of SOCS3 in the development of COPD.

For this purpose, we studied smokers with and without COPD and non-smokers’ lung tissue and BAL EVs for the presence of SOCS3 and its relation to the severity of disease and lung inflammation.

## Materials and methods

### Subject characteristics

Two groups (Group A and Group B) of patients were required for the study.

In Group A, 44 subjects undergoing lung resection for appropriate clinical indication were recruited, of whom 19 were smokers with COPD (SC) [9 severe COPD (GOLD stage III–IV: FEV_1_ < 50% predicted) and 10 mild to moderate COPD (GOLD stage I–II: FEV_1_ > 50% predicted)], 16 were smokers without COPD (S), and 9 were nonsmoking (NS) subjects with normal lung function. The nine subjects with severe COPD had Lung Volume Reduction Surgery (LVRS) for severe emphysema while the rest of the subjects had lung resection for solitary pulmonary nodules. In this group, lung tissue was examined for SOCS3 expression and lung inflammation using immunohistochemistry.

In Group B, 25 subjects undergoing bronchoscopy for appropriate clinical indication were recruited for the analysis of the expression of SOCS3 and its regulatory miRNAs within EVs in the bronchoalveolar lavage (BAL). In this group, 9 were smokers with COPD (SC) (GOLD stage I–II: FEV_1_ > 50% predicted), 11 were smokers without COPD (S), and 5 were nonsmoking (NS) subjects with normal lung function.

Patients underwent clinical evaluation comprising pulmonary function tests before surgery. COPD was defined as FEV_1_/FVC less than 70% after bronchodilator (26). None of the patients had past or present history of asthma, and no subject had an exacerbation of the disease.

### Study of SOCS3 expression and its relation to inflammation in lung tissue

Lung tissue preparation and immunohistochemistry were performed as previously described (27–29). Briefly, lungs were fixed in 4% formaldehyde and 5-µm-thick sections were cut and processed for morphometric and immunohistochemical analysis (details of the method in the online data supplement).

To quantify SOCS3 and TNFα expression in alveolar macrophages, at least 20 non-consecutive high-power fields (hpf) and at least 100 macrophages were evaluated for each subject. Results were expressed as percentage of SOCS3^+^ macrophages and percentage of TNFα^+^ macrophages over the total number of macrophages examined. To quantify CD8^+^ T lymphocytes in alveolar walls, at least 10 nonconsecutive fields were evaluated, and results were expressed as number of CD8^+^ cells per mm of alveolar wall (details of the method in the online data supplement).

Negative control for nonspecific binding done by either omitting the primary antibody or using isotype IgG revealed no signal. All analyses were performed with a Leica light microscope and a video recorder linked to a computerized image analysis system (LeicaLAS w3.8).

### Study of SOCS3 and its regulatory miRNAs in BAL-EVs

BAL was obtained and immediately processed (30, 31) (Group B). According to international guidelines, the BAL collection procedure consisted in positioning the bronchoscope in a wedged position within a bronchial opening. Subsequently, 200 mL of normal saline solution (divided into four aliquots of 50 mL each) at room temperature was instilled and retrieved. The average return ranged from 55 to 90 mL. BAL fluid was collected in a sterile tube for laboratory analysis.

### SOCS3 in EVs

BAL samples were filtered by a gauze filter (50-mm-size pore) to remove any mucus and centrifuged at 350*g* for 10 min at room temperature to separate supernatant from BAL cells. The BAL supernatants were centrifuged at 10,000*g* for 30 min at 4°C to isolate EV pellets. Finally, EVs were resuspended in ultrafiltered PBS and stored at −80°C. Flow cytometry analysis of EVs from BAL was performed using a CytoFLEX flow cytometer (Beckman-Coulter, USA) for the characterization and analysis of the EVs as previously reported (30, 31). Samples were then incubated with fluorescent-conjugated monoclonal antibodies for analysis. Macrophage-derived EVs were identified using CD14-APC (allophycocyanin), and SOCS3 surface expression on EVs was detected using SOCS3^+^ PE (phycoerythrin, both eBioscience, USA) (details of the method in the online data supplement). EV absolute count was expressed as events/microliter of the volume measured by the CytoFLEX. Files were evaluated by CytExpert Software (Version 2.3, Beckman-Coulter).

### miRNA in EVs

The miRNeasy MicroKit (Qiagen, Germany) was used for the purification and extraction of miRNAs from EV from BAL of all subjects. MiScript Primer Assays specific for has-miRNA-19a and has-miRNA-221 were used. miRNA expression was calculated using the Delta threshold cycle (Ct) method and normalized to *Caenorhabditis elegans* miRNA-39 (Cel-miRNA-39) and reported as the fold change determined by the comparative Ct method using Cel-miRNA-39 as internal control (32) (details of the method in the online data supplement).

The study conformed to the Declaration of Helsinki, and all patients provided informed written consent before surgery. All aspects of this study were approved by the local ethics committee (reference no. 0006045).

### Statistical analysis

Cases were coded, and measurements were made without knowledge of clinical data. Patient’s characteristics were expressed using the mean ± SD for clinical data and median (range) for morphological data. For continuous variables, normal distributions were tested using the Shapiro–Wilk test. One-way ANOVA with Tukey’s multiple comparison was used for the parametric clinical data, and the Kruskal–Wallis test and Dunn test for multiple comparison were used to evaluate the non-parametric morphological data and the differences among groups. Correlation coefficients were calculated using the nonparametric Spearman rank method. All data were analyzed by SPSS statistical software (version 3.5.2). *p* < 0.05 was considered statistically significant.

## Results

### Study of SOCS3 expression and its relation to inflammation in lung tissue (Group A)

**Table 1** shows the clinical characteristics of the subjects in Group A. The smoking history of smokers with and without COPD was similar. As expected, smokers with COPD had a lower value of FEV_1_ and FEV_1_/FVC (%) than smokers without COPD (*p* < 0.001) and nonsmokers (*p* < 0.001); 9/19 smokers with COPD were in GOLD stage 3 or 4, and 10 were in GOLD stage 1 or 2.

**Table 1:** Characteristics of the Group A subjects.

	Smokers with COPD (SC; n = 19)	Smokers without COPD (S; n = 16)	Non-smokers (NS; n = 9)	p
Age, years	67 ± 9	67 ± 9	69 ± 4	NS
Smoking history, pack-years	47 ± 21	39 ± 23	–	NS
Smoking status, current/ex	10/9	4/12	–	NS
FEV_1%_ pred	60 ± 31*	97 ± 11	98 ± 19	0.001
FEV_1_/FVC %	51 ± 17*	78 ± 6	79 ± 4	0.001

Values are expressed as mean ± SD.

The statistical analysis was performed using one-way ANOVA with Tukey as a post-hoc test.

*Significantly different from smokers without COPD and non-smokers (p < 0.001). NS: non-significant. –, not applicable.

**Figure 1:**
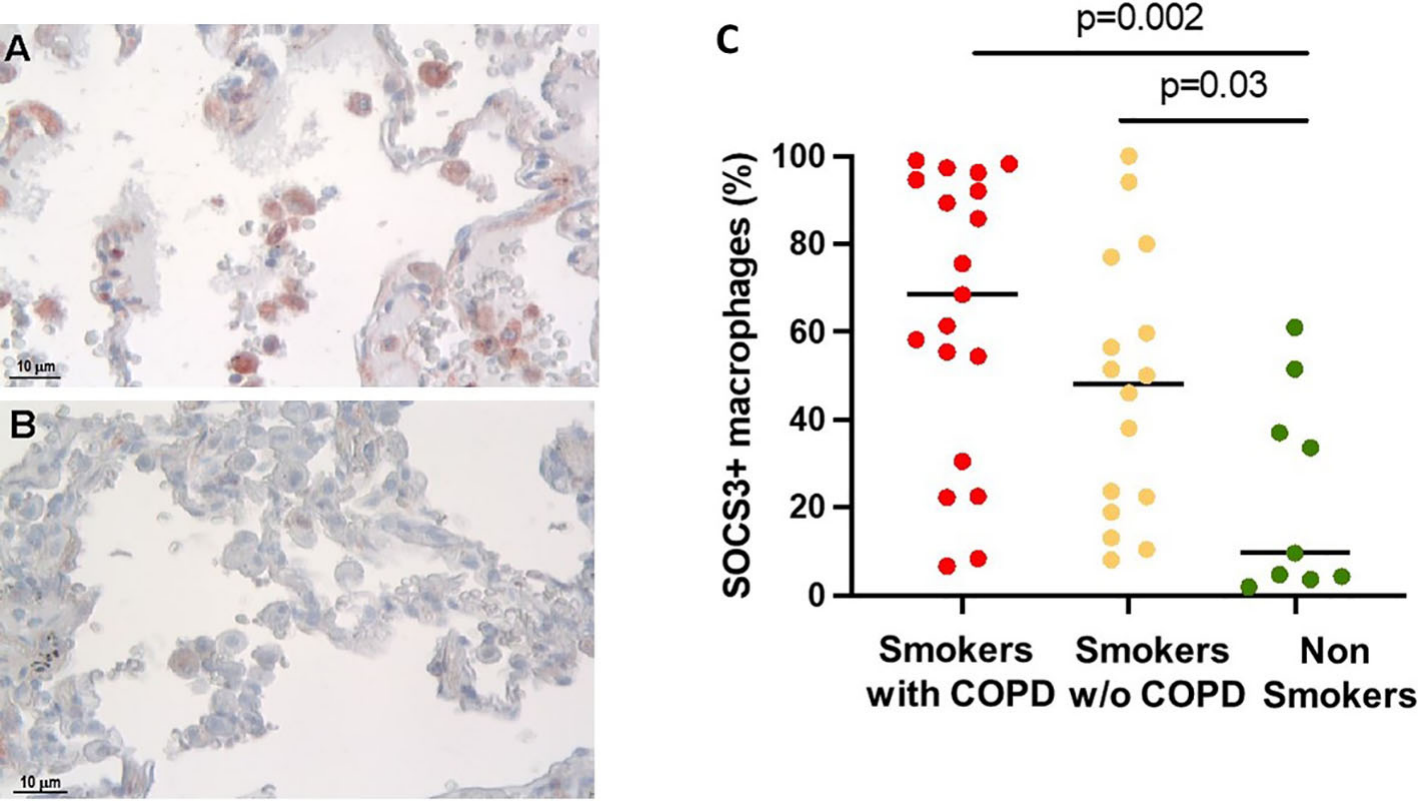
SOCS3 expression in alveolar macrophages (AM). **(A)** SOCS3-positive alveolar macrophages in smokers with COPD and **(B)** non-smokers. SOCS3 immunoreactivity appears as a red diffuse cytoplasmic granular pattern. Scale bars: 10 μm. **(C)** Percentage of AM SOCS3^+^ in smokers with COPD (red), smokers without COPD (yellow), and non-smokers (green). Horizontal bars represent median values. *p* < 0.001 by Kruskal–Wallis test with Dunn’s multiple comparison test.

The percentage of alveolar macrophages (AM) staining positively for SOCS3 (**Figures 1A, B**) was higher in smokers with [68 (6.6–99)%] and without COPD [48 (8–100)%] than in non-smokers [9.6 (1.9–61)%, *p* = 0.002 and *p* = 0.03 respectively, **Figure 1C**].

**Figure 2:**
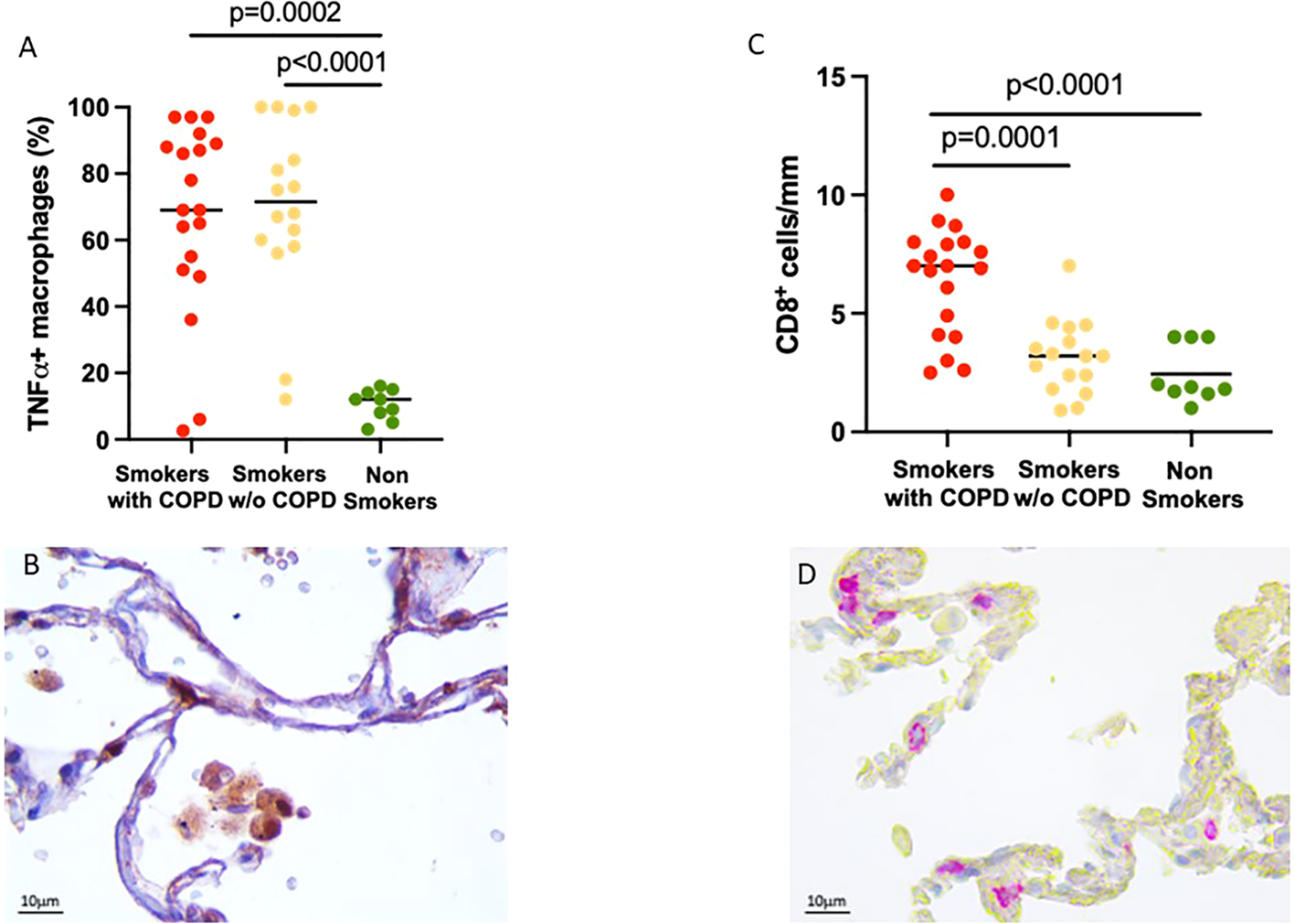
Evidence of lung inflammation: TNFα expression in alveolar macrophages and CD8^+^ T cell infiltration in alveolar walls. **(A)** Percentage of TNFα^+^ AM in smokers with COPD (red), smokers without COPD (yellow), and non-smokers (green). Horizontal bars represent median values. *p* < 0.001 by Kruskal–Wallis test with Dunn’s multiple comparison test. **(B)** TNFα^+^ AM in smokers with COPD. TNFα immunoreactivity appears as a brown diffuse cytoplasmic granular pattern. Scale bars: 10 μm. **(C)** Number of CD8+ T cells/mm of alveolar wall in smokers with COPD (red), smokers without COPD (yellow), and non-smokers (green). Horizontal bars represent median values. *p* < 0.001 by Kruskal–Wallis test with Dunn’s multiple comparison test. **(D)** Immunohistochemistry of CD8+ cells in the alveolar walls in smokers with COPD. CD8 immunoreactivity appears as a diffuse red color at the cytoplasmic level. Scale bars: 10 μm.

To verify the inflammatory phenotype of these AM, we looked at their expression of TNFα, which was higher in smokers with [70 (2–100)%] and without COPD [72 (12–100)%] compared to non-smokers [11 (1–19)%, *p* = 0.0002 and *p* < 0.0001 respectively, **Figures 2A, B**].

Further evidence of the important inflammatory state was the number of CD8^+^ T cells in the alveolar walls that was increased in smokers with COPD [7 (2.5–10) cells/mm] compared to smokers without COPD [3 (1–7) cells/mm, *p* = 0.0001, **Figure 2B**] and non-smokers [1.9 (1–4) cells/mm, *p* < 0.0001; **Figures 2C, D**].

**Figure 3:**
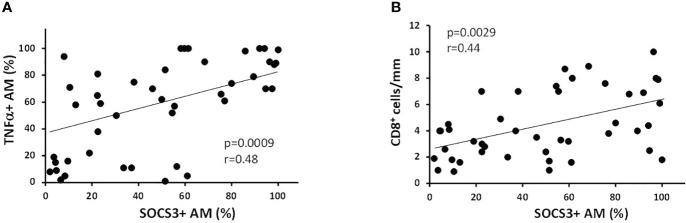
Correlation of % of SOCS3^+^ AM with inflammatory indexes. **(A)** The percentage of SOCS3^+^ AM was positively correlated with % of AM expressing TNFα (*r* = 0.48 and *p* = 0.0009) and **(B)** with the number of CD8^+^ cells/mm of alveolar wall (*r* = 0.44 and *p* = 0.0029). Spearman rank correlation test for both.

When all the subjects were considered together, the percentage of SOCS3^+^ AM were positively correlated with the amount smoked, expressed as pack-years (*r* = 0.39; *p* = 0.009), TNFα^+^ AM (*r* = 0.48; *p* = 0.0009, **Figure 3A**), and CD8^+^ cells per mm of alveolar wall (*r* = 0.44; *p* = 0.0029; **Figure 3B**).

### Study of SOCS3 and its regulatory miRNAs in BAL-EVs (Group B)

**Table 2** shows the characteristics of the subjects in Group B. There were no differences in age, amount smoked, and FEV_1_ and FEV_1_/FVC between the Group B and Group A subjects.

**Table 2:** Characteristics of the Group B subjects.

	Smokers with COPD (SC; n = 9)	Smokers without COPD (S; n = 11)	Non-smokers (NS; n = 5)	p
Age, years	70 ± 6	70 ± 6	62 ± 7	NS
Smoking history, pack-years	41 ± 12	42 ± 16	–	NS
Smoking status, current/ex	4/5	8/3	–	NS
FEV1% pred	54 ± 11*	100 ± 6	101 ± 11	0.001
FEV1/FVC %	55 ± 12*	84 ± 10	86 ± 5	0.001
Total cell counts (10^6^ mL)	3.2 ± 1.8	3.0 ± 1.7	3.1 ± 1.9	NS
Macrophages (%)	96.6 ± 1.4	96.5 ± 1.3	96.4 ± 1.6	NS
Neutrophils (%)	2.0 ± 1.1	1.9 ± 1.2	2.0 ± 1.6	NS
Lymphocytes (%)	1.4 ± 0.7	1.6 ± 0.7	1.6 ± 0.5	NS

* Values are expressed as mean ± SD.

The statistical analysis was performed using one-way ANOVA with Tukey as a post-hoc test.

*Significantly different from smokers without COPD and non-smokers (p < 0.001). NS: non-significant. –, not applicable.

The total number of EVs (events/μL) was similar in nonsmokers (4,297 ± 1,969 EVs/μL), smokers without COPD (3,786 ± 913 EVs/μL), and smokers with COPD (4,728 ± 2,837 EVs/μL). In 85% of the cases, the dimension of the EVs ranged between 200 and 500 nm and there was no difference between the three groups regarding EV size (*p* = 0.341).

The number of EVs expressing CD14 (a macrophage marker) was significantly higher in both smokers with COPD [610 (200–900) events/μL; *p* < 0.001] and those without COPD [360 (70–590) events/μL; *p* = 0.007] than in non-smokers [190 (110–298) events/μL]. They were also higher in smokers with COPD than in smokers without COPD (*p* = 0.046).

**Figure 4:**
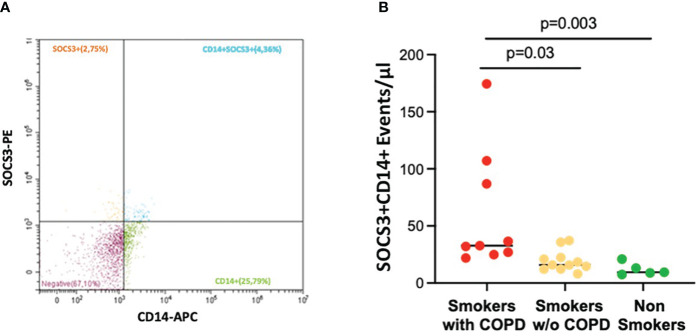
AM CD14^+^SOCS3^+^ EVs in BAL. **(A)** Representative EV profiles in BAL sample of a smoker with COPD, flow cytometric scatter plots. PE, Phycoerythrin; APC, allophycocyanin. **(B)** The number of events/μL of AM-derived EVs positive for SOCS3 was significantly higher in smokers with COPD (red) than in smokers without COPD (yellow) and non-smokers (green). Horizontal bars represent median values. *p* < 0.001 by Kruskal–Wallis test with Dunn’s multiple comparison test.

Macrophage-derived EVs expressing SOCS3^+^ (CD14^+^SOCS3^+^) were increased in smokers with COPD [33 (21–174) events/μL] compared to smokers without COPD [16 (8–37) events/μL; *p* = 0.03] and non-smokers [9 (7–21) events/μL; *p* = 0.003, **Figures 4A, B**].

**Figure 5:**
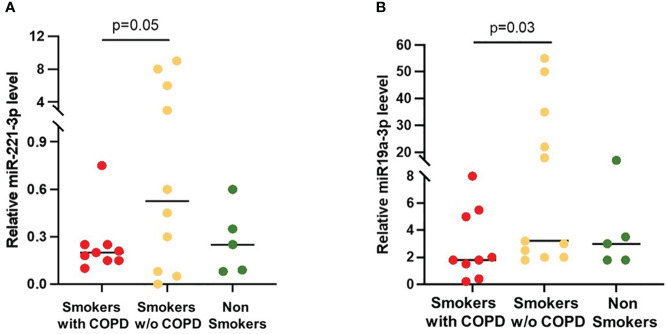
miRNAs expression in AM-derived EVs. **(A)** miRNA-221-3p and **(B)** miRNA-19a-5p levels were significantly higher in smokers without COPD (yellow) than in smokers with COPD (red). Horizontal bars represent median values. *p* < 0.001 by Kruskal–Wallis test with Dunn’s multiple comparison test.

The expression of the SOCS3 recognized regulators miRNA-19a-3p and miRNA-221-3p in BAL EVs was increased in smokers without COPD when compared to smokers with COPD [19 (2–53) *vs*. 3 (0.6–8), *p* = 0.03 and 3 (0.005–9.6) *vs*. 0.2 (0.08–0.7), *p* = 0.05, respectively; **Figures 5A, B**]. In non-smoking controls, miRNA-19a-3p [3 (2–20)] and miRNA-221-3p [0.25 (0.01–0.6)] expression was similar to those in COPD.

## Discussion

Studies in different animal models have proven the critical importance of SOCS3 in restraining inflammation and allowing optimal levels of protective immune responses against infections (33). However, the role of SOCS3 in inflammatory human diseases is controversial since it does not seem to have the same effects compared to that in the experimental models.

In an attempt to better define the role of SOCS3 in human diseases, we investigated the expression of this protein in the lung alveolar macrophages (AM) and in BAL EVs of smokers. SOCS3 expression in tissue AM was higher in smokers with and without COPD than in non-smokers, while SOCS3 expression in BAL AM-derived EVs (SOCS3^+^CD14^+^ EVs) was higher in smokers with COPD than in those without COPD and in non-smokers. Of interest, the levels of expression of the SOCS3 regulators miRNA-19a-3p and miRNA-221-3p, in BAL EVs, were increased in smokers without COPD when compared to COPD, whose level was similar to that of the non-smokers.

The finding of high expression of SOCS3 in tissue and BAL of smokers with COPD, in which a severe chronic adaptive immune lung inflammation plays a major role, is not easily conciliated with the described anti-inflammatory role of SOCS3 *in vitro* and in animal experiments. In our population, not only was SOCS3 highly expressed in smokers AM, but the degree of expression was directly related to the degree of lung inflammation as indicated by the numbers of CD8^+^ T cells in alveolar walls and the TNFα expression in alveolar macrophages. Similarly, a high expression of SOCS3 in inflamed tissue has been described in human IBD (16). Moreover, intestinal T cells from patients with IBD showed constitutive activation of STAT3 as well as SOCS3, which makes the function of SOCS3 in T cells in IBD “rather mysterious” (34). Activation of STAT3 has also been clearly described in human COPD and in smoking-induced COPD in mice (35, 36).

How could the simultaneous activation of STAT3 and SOCS3 be conciliated? Many of the cytokines such as IL-6 and other interleukins (IL) and interferons, critical orchestrators of persistent inflammatory states, including COPD, activate the Janus kinase/signal transducer and activator of transcription (JAK/STAT) pathway, which activates the transcription of downstream genes mediating the diverse effects of STAT3 in development and disease (37, 38). Dysregulation of the JAK-STAT pathway has been implicated in inflammatory disorders and cancer, further highlighting the necessary and vital role of SOCS3 in regulating the initiation, duration, and magnitude of cytokine signaling. Activation of STAT3 by tyrosine phosphorylation, an essential step for a normal inflammatory response, is kept in check by SOCS3, the major negative regulator of IL-6-dependent signaling, which is highly induced by activated STAT3, strongly inhibiting further STAT3 phosphorylation to prevent excessive activation of potentially deleterious gene expression (38).

This linear functional model, well described *in vitro* and in vertebrate to invertebrate animals, does not seem to function in humans, where even in the continued presence of SOCS3, STAT3 is rephosphorylated in response to IL-6 (38, 39). Rephosphorylation of STAT3 might occur because the interaction between SOCS3 and the IL-6 receptor is somehow prevented, or because continued STAT3 phosphorylation occurs through a mechanism not related to SOCS3. It is by now well recognized that the mechanism that explains the biphasic pattern of STAT3 activation in response to IL-6 is due to the participation of other receptors like EGFR, PDGFR, and Src kinase, and associated transcription factors like EGR-1 (39–41). Essentially, STAT3 phosphorylation can be sustained in the presence of functional SOCS3 if it is catalyzed by a receptor that is not inhibited by SOCS3, like EGFR and others. Aberrant regulation of STAT3 phosphorylation induces the expression of a subset of IL-6-dependent genes that would contribute importantly to diseases, involving altered regulation of immunity or cell proliferation and cancer (36, 40–42).

It has been shown that, in cigarette smoke-exposed primary bronchial epithelial cells from patients with COPD, EGFR activation regulates IL-6 receptor (42–44). Those studies have been corroborated by studies in human lungs in which EGFR expression, while weak or absent in healthy subjects (37–41), was increased in smokers and ex-smokers with COPD (44, 45) when compared to those without COPD. These findings support the relevance of the EGFR pathway in COPD development and progression. The presence of activated EGFR in the lungs of smokers could explain the high expression of phosphorylated STAT3 found in COPD (36), which would sustain the IL-6 roles in the acute phase response, chronic inflammation, autoimmunity, endothelial cell dysfunction, and cancer progression even in the presence of high levels of SOCS3 (36, 43).

In a different group of smokers and non-smokers with similar clinical characteristics to the original group, we have studied in BAL the presence of macrophage-derived EVs carrying on their surface SOCS3 (SOCS3^+^CD14^+^ EVs).

Alveolar macrophages not only express SOCS3 but also have the capacity, not shared by other inflammatory cells, to secrete SOCS3 in EVs (10–12). Smokers with COPD showed a significant increase in total CD14^+^SOCS3^+^ EVs when compared with those without COPD and non-smokers. We have previously described that alveolar macrophage-derived (CD14^+^) EVs are increased in COPD patients’ BAL when compared to smokers without COPD (30, 31), but the presence of SOCS3^+^ in macrophage-derived EVs in BAL of COPD patients had not been described before. Importantly, the reported increased counts of CD14^+^SOCS3^+^ EVs in COPD are not only due to the increased of total EVs but also due to the larger percentage of EVs expressing SOCS3 on their surface. Our finding adds to previously published data showing, in a small group of subjects, that SOCS3 in BAL was significantly decreased in smokers without COPD when compared to non-smokers (11). The production of SOCS3^+^CD14^+^ EVs by macrophages might be fundamental since they can communicate to other cells’ signals capable of suppressing STAT3 activation as it has been shown by Bourdonnay et al. (11). Our BAL results parallel the histological findings in tissue macrophages in a different group of patients, strengthening the significance of the results. It is possible that, in smokers without COPD, even if AM are producing SOCS3, as we have shown, the secretion of SOCS3 in EVs is somehow blocked, as our results comparing SOCS3 expression in AM and in EVs in this group seem to suggest.

In an attempt to understand the control of SOCS3 expression in COPD, we studied in BAL EVs the expression of miRNAs (miRNA-19a and miRNA-221), both recognized regulators of SOCS3 (17–24). Endothelial-derived EVs in plasma of smokers with COPD carry high levels of different miRNA as compared to healthy non-smokers. However, each EV is supposed to carry different cytokines and miRNAs depending on the original cell and context (homeostasis/inflammation). Thus, the study of miRNA in EVs derived from BAL macrophages, rather than EVs from plasma, ought to be the correct procedure to assess miRNAs’ presence and function with respect to the fate of SOCS3 in macrophages. Based on this knowledge, we assessed the presence and levels of miRNA-19a and miRNA-221 in BAL macrophage EVs and showed that while smokers without COPD had high levels of both miRNA19 and miRNA221, tuning down EV SOCS3 expression, smokers with COPD had a significantly lower miRNA expression, probably trying, ineffectively, to maintain high levels of SOCS3 to tune down STAT3 expression. The link between SOCS3 expression and miRNA levels in in BAL macrophage-derived EVs has not been studied before.

The downregulation of SOCS3 by both miRNA-19a and miRNA-221-3p has been shown to have untoward effects in cell proliferation and cancer development (17–24). As an important tumor suppressor, SOCS3 disfunction plays a catalytic role in the occurrence, progression, and metastasis of various tumors, such as colorectal cancer, gastric cancer, and thyroid cancer (46). The expression of SOCS3 might have an impact on the high-risk smokers have for the development of cancer, in the lungs and elsewhere (47). Of interest, we have shown that the prevalence of lung cancer in smokers is inversely related to the severity of COPD, thus greater prevalence in the least severe disease (47). It could be hypothesized, based on our results, that the suppression of SOCS3 in smokers with mild and/or no COPD might play a role in the development of cancer in this population (48).

The presence of lung cancer in our population could be a possible limitation to our study. However, half of the subjects with COPD had lung resection for volume reduction surgery for the treatment of emphysema and no cancer, with results that are in keeping with the rest of the group with cancer. The 10,000*g* centrifugation of the BAL fluid done to separate the EVs might result in a loss of smaller EVs/exosomes (50–150 nm) and miRNA associated with these EVs. This fact, along with using only one method to analyze the EVs, could be seen as a limitation of the study.

Finally, we have relied on the published evidence, and have not investigated the possible expression of EGFR and other receptors, which may promote the aberrant regulation of STAT3 phosphorylation in the cases in our study. Furthermore, the fact that the population for the study of EVs was different from the surgical population, even if these subjects were clinically and physiologically matched as closely as possible to the surgical cases, could be considered a limitation.

In conclusion, the suppressor function of SOCS3 in COPD seems to be overridden by other factors and does not follow the animal-model paradigm. Expression of SOCS3 in BAL macrophage-derived EVs might be useful to assess the degree of inflammation and possible progression of COPD. MiRNA downregulation of SOCS3 in smokers without COPD might contribute to the risk of developing cancer in these patients.

## Data availability statement

The original contributions presented in the study are included in the article/**Supplementary Material**. Further inquiries can be directed to the corresponding author.

## Ethics statement

The studies involving humans were approved by local ethics committee Azienda Ospedale -Università di Padova. The studies were conducted in accordance with the local legislation and institutional requirements. The participants provided their written informed consent to participate in this study.

## Author contributions

MT: Conceptualization, Data curation, Writing – original draft. ElB: Conceptualization, Data curation, Writing – original draft. SC: Formal analysis, Methodology, Writing – original draft. TN: Formal analysis, Methodology, Writing – original draft. DB: Investigation, Software, Supervision, Writing – original draft. MC: Formal analysis, Methodology, Software, Writing – original draft. AC: Methodology, Software, Writing – original draft. NB: Methodology, Writing – original draft. EC: Methodology, Software, Writing – original draft. GT: Investigation, Methodology, Writing – review & editing. SB: Methodology, Supervision, Writing – review & editing. AC: Supervision, Writing – review & editing. PS: Supervision, Writing – review & editing. MC: Conceptualization, Writing – review & editing. MS: Writing – review & editing. ErB: Conceptualization, Supervision, Writing – original draft, Writing – review & editing.
