# Supplementary material for: Suppressor of cytokine signaling-3 expression and its regulation in relation to inflammation in Chronic Obstructive Pulmonary Disease
Source: Front Immunol. 2024 Mar 12;15:1320077. doi: 10.3389/fimmu.2024.1320077 (PMC10963451; doi:10.3389/fimmu.2024.1320077)
Supplement: Supplementary file 1 [file DataSheet_1.pdf]

Suppressor of Cytokine Signaling 3 expression and its regulation in relation to inflammation in Chronic Obstructive Pulmonary Disease.

Mariaenrica Tinè*[1], Elisabetta Balestro*[1], Sara Carpi[2,3], Tommaso Neri[4], Davide Biondini[1,5], Maria Conti[1], Alvise Casara[1], Nicol Bernardinello[1], Elisabetta Cocconcelli[1], Graziella Turato[1], Simonetta Baraldo[1], Alessandro Celi[4], Paolo Spagnolo[1], Manuel G Cosio[1,6], Marina Saetta†[1], Erica Bazzan†[1]

ONLINE DATA SUPPLEMENT

Tissue Preparation and Histology

Selected tissue blocks (template size 1 × 2 × 2 cm) were taken from the subpleural parenchyma of the lobe obtained at surgery, avoiding areas involved by tumor, in subjects with mild to severe COPD, smokers without COPD and non-smokers. Samples were fixed in 4% formaldehyde in phosphate-buffered saline (PBS) at pH 7.2 and, after dehydration and embedded in paraffin wax. Tissue specimens were oriented, and 5-µm–thick sections were cut for immunohistochemical analysis. All sections were deparaffinated in xylene, hydrated through an alcohol series, washed twice in PBS.

Immunohistochemical Analysis

SOCS-3. Sections were incubated in 0.3% H2O2/PBS to quench endogenous peroxidase activity, immersed in citrate buffer 5 mM at pH 6.0, incubated in a microwave oven on high power for 30 min to epitope retrieval. The slides were then incubated overnight at 4° C with the primary mouse monoclonal antibody anti-SOCS3 diluted in blocking solution in a humidified chamber (Table S1). After five washes with PBS, sections were incubated with a goat anti-rabbit biotinylated secondary antibody (Vector Laboratories Inc., Burlingame, CA) followed by a preformed peroxidase conjugated avidin–biotin complex (Vectastain ABC Elite kit; Vector). The staining was revealed with the EnVision Detection System and Peroxidase/diaminobenzidine. Nuclei were counterstained with hematoxylin, and then sections were dehydrated through an alcohol series and permanently mounted with nonaqueous mounting medium (Eukit).

TNF-a. Sections were incubated in 0.3% H2O2/PBS to quench endogenous peroxidase activity, immersed in Tris-EDTA buffer at pH 9.0, incubated in a microwave oven on high power for 15 min to epitope retrieval. The slides were then incubated at room temperature with the primary mouse monoclonal antibody anti-TNF-a diluted in blocking solution in a humidified chamber (Table S1). After five washes with PBS, sections were incubated with a goat anti-rabbit biotinylated secondary antibody (Vector Laboratories Inc., Burlingame, CA) followed by a preformed peroxidase conjugated avidin–biotin complex (Vectastain ABC Elite kit; Vector). The staining was revealed with the EnVision Detection System and Peroxidase/diaminobenzidine. Nuclei were

counterstained with hematoxylin, and then sections were dehydrated through an alcohol series and permanently mounted with with nonaqueous mounting medium (Eukit).

CD8+ T-lymphocytes. Sections were immersed in citrate buffer 5 mM at pH 6.0, incubated in a microwave oven on high power for 30 min to epitope retrieval. The slides were then incubated at room temperature with the primary mouse monoclonal antibody anti-CD8 diluted in blocking solution in a humidified chamber (Table S1). Monoclonal antibody binding was detected with the alkaline phosphatase, antialkaline phosphatase method (VECTASTAIN® ABC-AP Kit, Alkaline Phosphatase). The staining was revealed with the fast-red substrate. Nuclei were counterstained with hematoxylin, and then sections were dehydrated through an alcohol series and permanently mounted with aqueous mounting medium (Glycerol).

TABLE S1: details of immunohistochemical conditions

**Antibody**	**Dilution**	**Company**	**Antigen retrieval method**	**Antigen detection**
Polyclonal rabbit anti-SOC3 (ab53984)<br>Validated in IHC-P and tested in Human samples<br>(https://www.abcam.com/en-it/products/primary-antibodies/socs3-antibody-ab53984)	1:100 (overnight)	AbCam, UK	30' Microwave in citrate buffer pH 6	Envision detection system, peroxidase and diaminobenzidine (DAB)
Monoclonal rabbit anti-TNF-a (ab270264)<br>Validated in IHC-P and tested in Human samples.<br>https://www.abcam.com/en-it/products/primary-antibodies/tnf-alpha-antibody-tnf-1500r-ab270264	1:500	AbCam, UK	30' Microwave in Tris-EDTA buffer pH 9	Envision detection system, peroxidase and diaminobenzidine (DAB)
Monoclonal mouse anti-CD8 (M7103). Validated in IHC-P and tested in Human samples.<br>https://www.agilent.com/en/product/immunohistochemistry/antibodies-controls/primary-antibodies/cd8-(concentrate)-76631	1:10	DAKO, UK	30' Microwave in citrate buffer pH 6	VECTASTAIN® ABC-AP Kit, Alkaline Phosphatase and liquid permanent red

Negative Controls in Immunohistochemical Analysis

Negative control for SOCS3 was performed using the isotype control antibody from the same species (IgG rabbit). The section was then incubated with a goat anti-rabbit biotinylated secondary antibody (Vector Laboratories Inc., Burlingame, CA) followed by peroxidase conjugated avidin–biotin complex (Vectastain ABC Elite kit; Vector) and EnVision Detection System and Peroxidase/diaminobenzidine. Fig E1 relieved no SOCS3 positive staining in lung tissue.

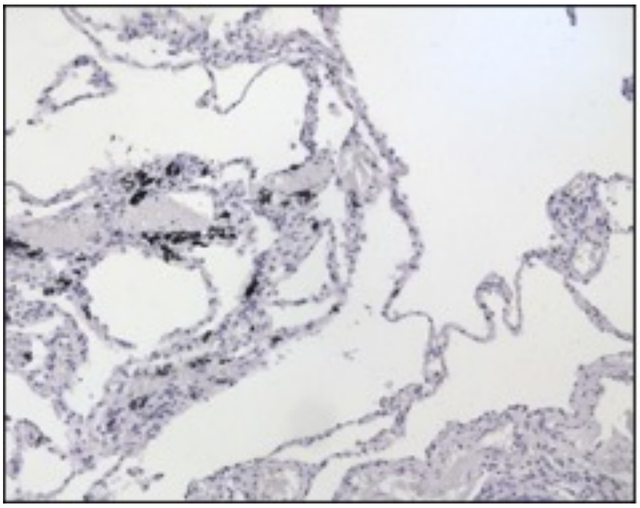

Fig E1: Negative control for SOCS3 staining.

Negative control for TNF-α was performed using the isotype control antibody from the same species (IgG rabbit). The section was then incubated with a goat anti-rabbit biotinylated secondary antibody (Vector Laboratories Inc., Burlingame, CA) followed by peroxidase conjugated avidin–biotin complex (Vectastain ABC Elite kit; Vector) and EnVision Detection System and Peroxidase/diaminobenzidine. Fig E2 relieved no TNF-a positive staining in lung tissue.

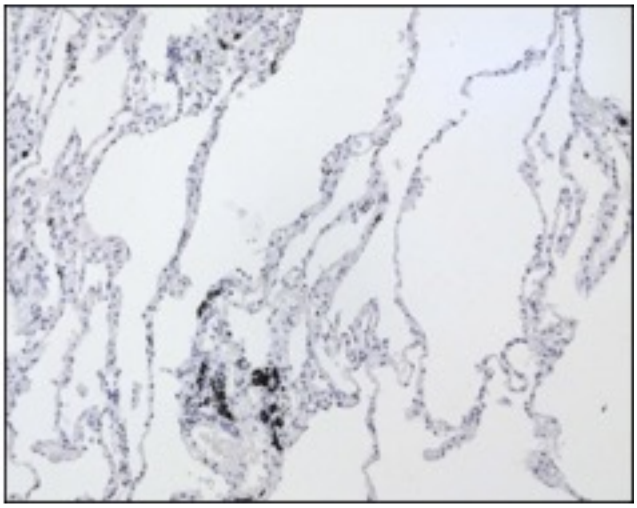

Fig E2: Negative control for TNF-a staining.

Negative control for CD8 TCells was performed omitting the primary antibody anti-CD8. The section was then incubated with the alkaline phosphatase, antialkaline phosphatase (VECTASTAIN® ABC-AP Kit, Alkaline Phosphatase) and fast-red substrate. Fig E3 relieved no CD8 positive staining in lung tissue.

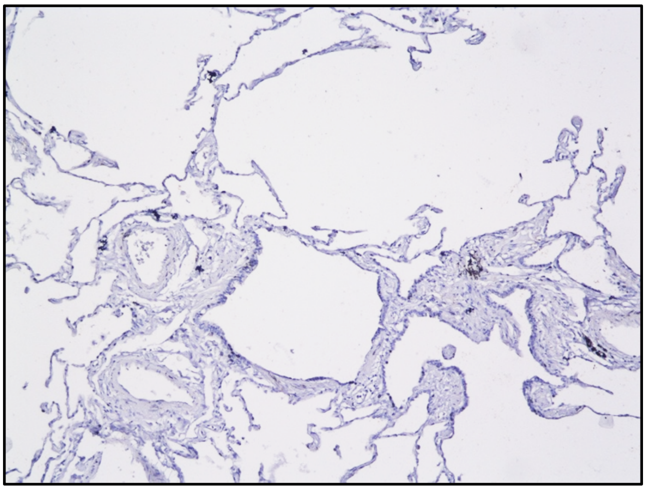

Fig E3: Negative control for CD-8 staining.

Positive macrophages in alveolar spaces

Alveolar macrophages were defined as mononuclear cells with well-represented cytoplasm present in the alveolar spaces and not attached to the alveolar walls. To quantify positive alveolar macrophages, at least 20 high-power fields (hpf) of lung parenchyma were randomly selected for each section and at least 100 macrophages inside alveoli were evaluated. Results were expressed as percentage of positive macrophages over total macrophages examined.

Positive cells in lung parenchyma

Analysis of lung parenchyma was performed using a light microscope (Leica DMLB; Leica, Cambridge, UK) connected to a video recorder linked to a computerized image system (Software: Leica Application Suite). The cases were coded, and the measurements made without knowledge of clinical data.

For the cell counts in the lung parenchyma, we examined only the alveolar walls with a single layer of cells to avoid bias caused by technical artifacts such as adjacent alveolar walls. The number of inflammatory cells within the alveolar walls was computed. Briefly, at a magnification ×630, we measured the length of the alveolar walls, and the number of positive cells within these alveolar walls was counted. Ten fields randomly distributed across the slide were studied per subject, and the result was expressed as the number of positive cells/mm of alveolar wall. We decided to examine 10 fields per subject since this number of fields was sufficient to obtain a mean value per subject that remained rather constant after further increasing the number of fields examined.

Study of SOCS3 and its regulatory miRNAs in BAL-EVs

BAL-EVs

Bronchoalveolar lavage (BAL) was obtained, and immediately processed.

BAL samples were filtered by a gauze filter (50-µm-size pore) to remove any mucus and centrifuged at 350 g for 10 min at room temperature, to separate supernatant from BAL cells. The BAL supernatants were centrifuged at 10,000 g 30 min at 4°C to isolate EV pellets. Finally, EVs were resuspended in ultrafiltered PBS and stored at 80°C (E1-E3).

The new generation of flow cytometer (CytoFLEX Beckman Coulter), which can detect EVs to a size as low as 100 nm, was used to identify and separate the EV population according to dimension, as suggested by minimal information for studies of extracellular vesicles (MISEV) guidelines (E3).
For EV size calibration of the flow cytometer, fluorescent polystyrene beads (Megamix FSC & SSC Plus, BioCytex, Marseille, France) were used in sizes of 0.1, 0.16, 0.2, 0.24, 0.3, 0.5, and 0.9 µm. Violet side scatter (VSSC) and FL1 channel gain were set to visualize the beads. The side scatter (SSC) from the 405 nm violet laser (VSSC) was used as a trigger signal to discriminate the noise. Megamix bead solution was gated, excluding the background noise (because of the solution itself). After turning the set in VSSC and forward scatter (FSC), a rectangular gate was set between the 0.1 µm and 0.9 µm bead to select particles that might be included in the range of exosomes and ectosomes and exclude larger vesicles such as apoptotic bodies, usually falling in the 1–4 µm range of size.
For the characterization and analysis of the EVs, 20 µL of samples were stained with 10 µL of calcein-AM (Sigma-Aldrich, Milan, Italy), to confirm the presence and integrity of EVs. Samples were then incubated in the dark for 30 min at room temperature with 2 µL of fluorescent-conjugated monoclonal antibodies against cell-type specific antigens. Stained samples were then diluted by adding 140 µL of sterile filtered PBS.
Macrophage-derived EVs were identified using CD14-APC (allophycocyanin, eBioscience, San Diego, CA, USA) and SOCS3 presence on EVs was detected using SOCS3+PE (phycoerythin, both eBioscience, USA). The incubation of samples with the appropriate isotype controls was subtracted from the positive antibody sample to avoid nonspecific signals. True EV events were defined as positive stained for: calcein-AM and anti-CD14 and anti-SOCS3.
EV absolute count was expressed as events per microliters of the volume measured by the CytoFLEX. Files were exported, and data were evaluated by CytExpert Software (Version 2.3, Beckman Coulter).

miRNA in EVs

The miRNeasy Mini Kit (Qiagen, Hilden, Germany) was used for purification and extraction of miRNAs from EV isolated from BAL of all subjects. The retro-transcription of extracted miRNAs was performed by using the miScript Reverse Transcription Kit (Qiagen) (E4). The qPCR experiments were performed by miScript SYBR-Green PCR kit (Qiagen), as previously reported (E5). Signals

were detected on the MiniOpticon CFX 48 real-time PCR Detection System (Bio-Rad, Hercules, CA, USA). MiScript Primer Assays specific for has-miRNA-19a and has-miRNA-221 were obtained from Qiagen. miRNA expression was calculated using the Delta threshold cycle (Ct) method and normalized to Caenorhabditis elegans miRNA-39 (Cel-miRNA-39) and reported as the fold change determined by the comparative Ct method using Cel-miRNA-39 as internal control.

References

E1. Carnino JM, Lee H, Jin Y. Isolation and characterization of extracellular vesicles from Broncho-alveolar lavage fluid: a review and comparison of different methods. Respir Res 20: 240, 2019.

E2. Lötvall J, Hill AF, Hochberg F, Buzás EI, Di Vizio D, Gardiner C, Gho YS, Kurochkin IV, Mathivanan S, Quesenberry P, Sahoo S, Tahara H, Wauben MH, Witwer KW, Théry C. Minimal experimental requirements for definition of extracellular vesicles and their functions: a position statement from the International Society for Extracellular Vesicles. J Extracell Vesicles 3: 26913, 2014.

E3. Théry C, Witwer KW, Aikawa E, Alcaraz MJ, Anderson JD, Andriantsitohaina R. Minimal information for studies of extracellular vesicles 2018 (MISEV2018): a position statement of the International Society for Extracellular Vesicles and update of the MISEV2014 guidelines. J Extracell Vesicles 7: 1535750, 2018.

E4. Adinolfi, B.; Carpi, S.; Romanini, A.; Da Pozzo, E.; Castagna, M.; Costa, B.; Martini, C.; Olesen, S.-P.; Schmitt, N.; Breschi, M.C.; et al. Analysis of the antitumor activity of clotrimazole on A375 human melanoma cells. Anticancer Res. 2015, 35, 3781–3786.

E5. Carpi, S.; Fogli, S.; Polini, B.; Montagnani, V.; Podestà, A.; Breschi, M.C.; Romanini, A.; Stecca, B.; Nieri, P. Tumor-promoting effects of cannabinoid receptor type 1 in human melanoma cells. Toxicol. In Vitro 2017, 40, 272–279
